# Acute abdomen due to spilled gallstones: a diagnostic dilemma 10 years after laparoscopic cholecystectomy

**DOI:** 10.1093/jscr/rjaa275

**Published:** 2020-08-24

**Authors:** Mehmet Tolga Kafadar, İsmail Çetinkaya, Ulaş Aday, Ömer Başol, Hüseyin Bilge

**Affiliations:** Dicle University School of Medicine, Department of General Surgery, Diyarbakır, Turkey; Health Sciences University, Mehmet Akif İnan Training and Research Hospital Clinic of General Surgery, Şanlıurfa, Turkey; Dicle University School of Medicine, Department of General Surgery, Diyarbakır, Turkey; Dicle University School of Medicine, Department of General Surgery, Diyarbakır, Turkey; Dicle University School of Medicine, Department of General Surgery, Diyarbakır, Turkey

## Abstract

Laparoscopic cholecystectomy (LC) carries a significant risk of gallbladder perforation and resulting scattering of bile stones into the abdominal cavity. The retrieval of the spilled stones is not always possible by laparoscopic technique. Most cases do not create long-term problems, although some cases may be complicated in future and create uncertainties regarding the correct diagnosis. Diagnosis can be difficult, and in most cases the patient may require open surgery for management of these complications. Herein, we report a case of acute abdomen due to spilled stones occurring 10 years after LC. In the first stage, definitive diagnosis could not be made with computed tomography examination. Finally, the patient was diagnosed with explorative laparotomy.

## INTRODUCTION

Laparoscopic cholecystectomy (LC) has become the standard treatment for symptomatic cholelithiasis and acute cholecystitis. However, LC is associated with some complications that are rare in open cholecystectomy [1]. Gallbladder perforation with intra-abdominal spillage of gallstones is a common complication during LC, although it is not considered serious. Laparoscopic bile stone retrieval is of limited success and most stones may remain in abdomen unless care is taken to retrieve them [2]. In this article, we present a 42-year-old female patient with acute abdominal pain and swelling due to lost gallstones from LC performed 10 years previously.

## CASE REPORT

**Figure 1:**
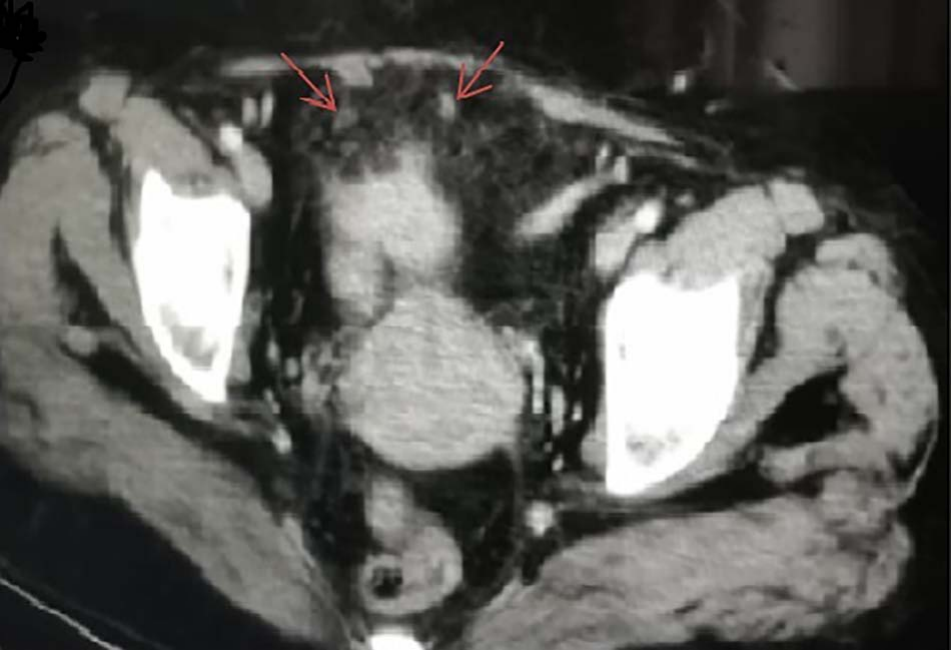
CT scan of abdomen and pelvis axial view showing intra-abdominal gallstones.

**Figure 2:**
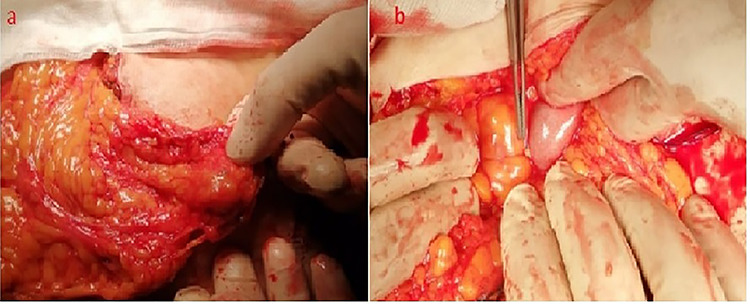
Intraoperative appearance of the spilled gallstones (**a**, **b**).

A 42-year-old woman presented to our emergency department with a painful swelling in the suprapubic region persisted for 3 days. She had a history of LC 10 years back at our center for symptomatic gallstones and had no history of comorbidities. The patient had a history of cesarean section twice. She had a pulse rate of 92/min, blood pressure of 110/60 and a temperature of 37.4°C. Physical examination revealed abdominal tenderness in the suprapubic region, right and left lower quadrant, and exhibited signs of peritoneal irritation, muscle guarding and rebound tenderness. Laboratory tests resulted with white blood cell: 15 200/mm^3^, hemoglobin: 11.9 g/dl, C-reactive protein: 24 mg/dl, and other biochemical parameters were also normal. On oral contrast-enhanced abdominal computed tomography (CT) performed in the emergency room (Fig. 1), the mesenteric adipose planes were inflamed and contaminated. Minimal free fluid was observed in the periphery of the intestinal loop in the pelvic area. At first, it was not stated on tomographic interpretation that there were gallstones in the abdomen. Considering that the patient had signs and symptoms of acute abdomen, she underwent diagnostic laparoscopy. In exploration, it was observed that the small intestines were edematous, the omentum was inflamed in the pelvic region and the omentum was attached to the anterior abdominal wall, bladder and uterus by gato. Since a clear diagnosis could not be made in the patient for etiology, abdomen was opened with a midline incision under the umbilicus. Infected reactive fluid located between the omentum and the anterior abdominal wall and pelvic region was aspirated. Adhesions due to previous cesarean sections were removed. During the adhesiolysis, stones the largest of which was ~2 cm in size, and abscesses were detected in the omental granuloma/cake (Fig. 2a, b). These stones were thought to remain in the abdomen due to the previous LC. Partial omentectomy with abscesses drainage was performed. The abdomen was irrigated and the stones were retrieved. No other pathology was detected in exploration and no additional surgical intervention was performed. The postoperative period was uneventful, and she was discharged on the fourth postoperative day. Spilled cholesterol gallstones were determined to be the cause of the acute abdomen in this case. Histopathological examination confirmed the diagnosis. Written informed consent was obtained from the patient for the anonymized information to be published in this article.

## DISCUSSION

Despite the rare nature of gallbladder perforation and resulting scattering of bile stones into the abdominal cavity, they may still cause diagnostic challenges and substantial morbidity in both early and late postoperative periods. Complications due to peritoneal gallstones after LC are infrequent with a rate of 1.7 complications per 1000 cases [3]. The reported incidence of gallbladder perforation is 10–40%, that of gallstone spillage is 7.3% and that of unretrieved peritoneal gallstones is 2.4% [4]. In a report on 10 174 patients, stones spilled in 581 patients (5.7%) and were retrieved in only 34 patients. In the remaining 547 patients, only 8 patients (0.08%) developed complications [5].

Stone spillage from gallbladder occurs during gall bladder extraction, by tearing with grasping forceps or during dissection of the gallbladder from the liver bed. Predisposing factors for gallbladder perforation and stone spillage include acute cholecystitis, pericholecystic adhesions, obesity and male sex. The problem of stone spillage is aggravated by spread of calculi because of peritoneal irrigation and pneumoperitoneum [6].

The reported complications due to gallstone spillage include peritoneal–cutaneous sinus tracts, intra-abdominal abscess, liver abscess, subhepatic inflammatory mass, persistent discharging trocar sites, micro abscesses, retroperitoneal abscess, granuloma, cholelithoptysis, small bowel obstruction, small bowel fistula, colonic fistula, bowel perforation and ileus as a result of sticking of small intestine inserts into the abscess wall. Bile stones may remain silent in the abdominal cavity for a long time until a septicemic focus causes calculus to become infected and produce abscesses [7].

Although early complications often occur in the form of peritonitis or abscesses, late complications usually occur as a result of migration of the stones. The organism tries to throw the stone out of it by accepting it as a foreign body. Generally, complications require surgical intervention. Such patients should be well informed after surgery, and stone spillage should be well documented in order to avoid medico-legal issues and diagnostic dilemmas in the future. In our case, no documentation or surgical notes about previous surgeries were found. Complications due to gallstone spillage may take months or years to present. Ultrasound, CT or magnetic resonance imaging can be used to detect a spilled stone in suspected cases presenting late after cholecystectomy [8].

Radiological interventions, laparoscopic methods and open surgery are available to remove such stones from the abdominal cavity and drain any abscess if present. Abscess puncture and aspiration without stone removal should be avoided because these often result in recurrence. An observational study involving 82 surgeons revealed that conversion to an open procedure was performed only in 3% of patients in whom gall stones were spilled during LC. Although removal of spilled gallstones is not recommended for all patients, an attempt at removal should be performed whenever possible [9]. Our case exemplifies the fact that spilled gallstones can produce complications even many years after cholecystectomy.

Brockmann *et al*. [10] reported that the most frequent complication due to intraperitoneal gallstones is abscess formation, accounting for 60% of complications. In their report, the predisposing factors for the development of complications after gallstone spillage were older age, acute cholecystitis, chronic cholecystitis with thickened gallbladder wall, male sex, spillage of pigment gallstones, perihepatic localization of lost stones, previous laparotomy and number or size of stones. Retrieving spilled gallstones through vigorous peritoneal lavage is important to reduce the rates of subsequent complications. If complications occur, definitive treatment requires both drainage of any abscess and removal of the spilled gallstones.

## CONCLUSION

During LC, it is common to encounter gallbladder perforation ending up with spillage and loss of some gallstones. It is important to attempt to retrieve spilled gallstones. Though uncommon, these stones may lead to early or late complications, which can be a diagnostic challenge and cause significant morbidity to the patient.

## Conflict of interest statement

None declared.

## AUTHOR CONTRIBUTIONS

Concept: M.T.K., İ.Ç.; Design: M.T.K., U.A., Ö.B.; Supervision: M.T.K., Ö.B., H.B.; Fundings: M.T.K., İ.Ç.; Materials: M.T.K.; Data collection and/or processing: İ.Ç.; Analysis and/or interpretation: M.T.K., Ö.B., H.B.; Literature review: M.T.K., U.A., H.B.; Writing: M.T.K.; Critical review: M.T.K., U.A.

## INFORMED CONSENT

Written informed consent was obtained from the patient for the anonymized information to be published in this article.
